# Genome aliquoting with double cut and join

**DOI:** 10.1186/1471-2105-10-S1-S2

**Published:** 2009-01-30

**Authors:** Robert Warren, David Sankoff

**Affiliations:** 1 School of Information Technology and Engineering, University of Ottawa, 800 King Edward Avenue, Ottawa, Canada; 2 Department of Mathematics and Statistics, University of Ottawa, 585 King Edward Avenue, Ottawa, Canada

## Abstract

**Background:**

The *genome aliquoting probem *is, given an observed genome *A *with *n *copies of each gene, presumed to descend from an *n*-way polyploidization event from an ordinary diploid genome *B*, followed by a history of chromosomal rearrangements, to reconstruct the identity of the original genome *B'*. The idea is to construct *B'*, containing exactly one copy of each gene, so as to minimize the number of rearrangements *d*(*A, B' *⊕ *B' *⊕ ... ⊕ *B'*) necessary to convert the observed genome *B' *⊕ *B' *⊕ ... ⊕ *B' *into *A*.

**Results:**

In this paper we make the first attempt to define and solve the genome aliquoting problem. We present a heuristic algorithm for the problem as well the data from our experiments demonstrating its validity.

**Conclusion:**

The heuristic performs well, consistently giving a non-trivial result. The question as to the existence or non-existence of an exact solution to this problem remains open.

## Background

Occasionally, during evolution, a genome *B *will convert into an *n*-fold replicate of itself, creating a *polyploid B *⊕ *B *⊕ ... ⊕ *B*, containing *n *identical copies of every gene and every chromosome. Over time, through genome rearrangements, the sets of chromosomes are intermingled and the identity of the original genome is lost. Thus, brewers' yeast descends from an ancient tetraploid and wheat from an ancient hexaploid. Then the *genome aliquoting probem *is, given an observed genome *A *with *n *copies of each gene, to reconstruct the identity of the original genome *B'*.

The aliquoting problem is a generalization from the *genome halving problem *[1,2], just as polyploidization is a more general process than tetraploidization. High-order polyploids are particularly prevalent in plants. We will illustrate with a data set on hexaploid wheat *n *= 3.

The *genome halving problem *has been solved several times. The first solution to this problem was published in [1], which solved this problem with respect to inversion and translocation distance. [3] corrected a small problem in [1] with respect to unichromosomal genomes. In [2] the problem was solved with respect to the inversion, translocation and block interchange distance; an approach that was later refined in [4]. All of these algorithms are linear time and very efficient and proven to return the most parsimonious solution.

Until now, there have not been any algorithms to solve the more general genome aliquoting problem. Our algorithm for the genome aliquoting problem is an extension of the genome halving algorithms, primarily the algorithm from [2], to handle polyploids with three or more copies of every gene.

### Notation

In this section we introduce our notation for genomes. A gene *a *represents an oriented sequence of DNA whose two *extremities *are its *tail *$\overset{>-}{a}$ and its *head *$\vec{a}$. The *adjacency *of two consecutive genes *a *and *b *is denoted by an unordered set, either $\left\{\vec{a},\overset{>-}{b}\},(=\{\overset{>-}{a},\vec{a}\}),\{\vec{a},\vec{b}\},\{\overset{>-}{a},\overset{>-}{b}\},\{\overset{>-}{a},\vec{b}\right\}$, depending on the order and orientation of *a *and *b*.

An extremity that is not adjacent to any other extremity is called a *telomere *and is represented by a singleton set {$\vec{a}$} or {$\overset{>-}{a}$}. A *genome *is represented by an unordered set of adjacencies and telomeres such that the head and tail of each gene appear exactly once.

A *duplicated genome *is a genome with two or more copies of each gene such that the head and the tail of every gene appear exactly *p *≥ 2 times. To differentiate the genes we arbitrarily assign each gene a subscript. Thus, we say that gene *a *is a *unique gene *with *paralogs a*_1,_*a*_2_, ... *a*_*p *_with corresponding *paralogous extremities * and $\vec{a_{1}},\vec{a_{2}},...,\vec{a_{p}}$.

Without a loss of generality, we say that two adjacencies {*a*, *b*} and {*c*, *d*} are *compatible *if both *a *is paralogous with *c *and *b *is paralogous with *d *or if neither *a *nor *b *is paralogous with either of *c *or *d*. For example, $\left\{\overset{>-}{a_{1}},\vec{b_{3}}\right\}$ and $\left\{\overset{>-}{a_{2}},\vec{b_{2}}\right\}$ are compatible but neither is compatible with $\left\{\overset{>-}{d_{4}},\vec{b_{1}}\right\}$ but all three are compatible with $\left\{\vec{c_{2}},\overset{>-}{d_{4}}\right\}$. Any two telomeres are always compatible. A telomere and an adjacency are compatible if the telomere's extremity is not paralogous with either of the extremities in the adjacency. We say that a set of adjacencies and telomeres is compatible if no two elements of the set are incompatible.

**Definition 1 ***Let A be a duplicated genome. A is *valid *if and only if:*

• *If there exists an x and there exists a y such that *{*u*_*x*_, *v*_*y*_} ∈ *A and u*_*x*_*is not paralogous with v*_*y*_*then for all *1 ≤ *x *≤ *p there exists a y such that*{*u*_*x*_, *v*_*y*_} ∈ *A*

• *If there exists an x such that *{*u*_*x*_} ∈ *A then for all *1 ≤ *x *≤ *p *{*u*_*x*_} ∈ *A*.

**Figure 1:**
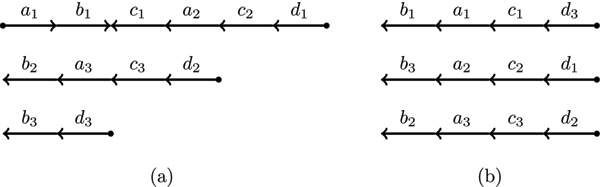
**Duplicated genomes**. Two different genomes both with three chromosomes, four unique genes each with three paralogs. (a) The rearranged duplicated genome represented by the unordered set $\left\{\{\overset{>-}{a_{1}}\},\{\vec{a_{1}},\overset{>-}{b_{1}}\},\{\vec{b_{1}},\vec{c_{1}}\},\{\overset{>-}{c_{1}},\vec{a_{2}}\},\{\overset{>-}{a_{2}},\vec{c_{2}}\},\{\overset{>-}{c_{2}},\vec{d_{1}}\},\{\overset{>-}{d_{1}}\},\{\overset{>-}{d_{2}}\},\{\vec{d_{2}},\overset{>-}{c_{3}}\},\{\vec{c_{3}},\overset{>-}{a_{3}}\},\{\vec{a_{3}},\overset{>-}{b_{2}}\}\{\vec{b_{2}}\},\{\vec{b_{3}}\},\{\overset{>-}{b_{3}},\vec{d_{3}}\},\{\overset{>-}{d_{3}}\}\right\}$. (b) The perfectly duplicated genome represented by the unordered set $\left\{\{\vec{b_{1}}\},\{\overset{>-}{b_{1}},\vec{a_{1}}\},\{\overset{>-}{a_{1}},\vec{c_{1}}\},\{\overset{>-}{c_{1}},\vec{d_{3}}\},\{\overset{>-}{d_{3}}\},\{\overset{>-}{b_{3}}\},\{\overset{>-}{b_{3}},\vec{a_{2}}\},\{\overset{>-}{a_{2}},\vec{c_{2}}\},\{\overset{>-}{c_{2}},\vec{d_{1}}\},\{\overset{>-}{d_{1}}\},\{\vec{b_{2}}\},\{\overset{>-}{b_{2}},\vec{a_{3}}\},\{\overset{>-}{a_{3}},\vec{c_{3}}\},\{\overset{>-}{c_{3}},\vec{d_{2}}\},\{\overset{>-}{d_{2}}\}\right\}$.

A duplicated genome that is valid is a *perfectly duplicated genome *(see Figure 1b for an example). Similarly, an invalid duplicated genome is called a *rearranged duplicated genome *(see Figure 1a for an example).

We can now define the problem:

**Definition 2 ***The *genome aliquoting problem *is defined as follows: given a rearranged duplicated genome A find a perfectly duplicated genome B such that the distance between A and B is minimal with respect to some distance metric*.

In this paper, the distance metric we will use is the *double cut and join distance*. The *distance *between two genomes is the shortest sequence of rearrangement operations needed to transform a genome *A *into a genome *B*. With double cut and join, the set of rearrangement operations used to compute the distance includes translocations, fusions, fissions, inversions and block interchanges (an approximation of a transposition). Double cut and join was introduced in [5] and refined later in [6]. It is the later paper from which we draw the following formal definition of double cut and join:

**Definition 3 ***The *double cut and join *operation acts on two adjacencies or telomeres u and v of a genome in one of the following three ways:*

• *If both u *= {*p*, *q*}*and v *= {*r*, *s*}*are adjacencies, these are replaced by the two adjacencies *{*p, r*}*and *{*s, q*}*or by the two adjacencies *{*p, s*}*and *{*q, r*}.

• *If u *= {*p*, *q*}*is an adjacency and v *= {*r*}*is a telomere, these are replaced by *{*p, r*}*and *{*q*}*or by *{*q, r*}*and *{*p*}.

• *If both u *= {*q*}*and v *= {*r*}*are telomeres, these are replaced by *{*q, r*}.

*In addition, as an inverse of the last case, a single adjacency *{*q, r*}*can be replaced by two telomeres *{*q*}*and *{*r*}.

## Methods

**Figure 2:**
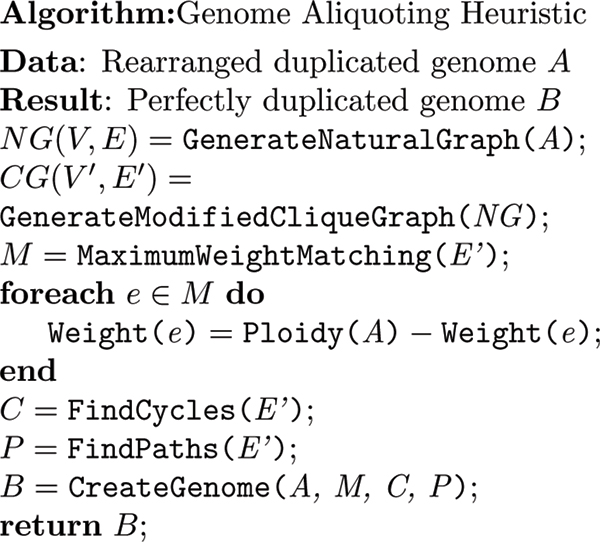
**Algorthim**. A pseudocode description of the genome aliquoting algorithm.

The pseudocode for the algorithm is given in Figure 2. In following sections we breakdown and explain the various steps of the algorithm in detail as well as our implementation of the algorithm.

### Generate natural graphs

Like when solving the genome halving problem, the first step for the genome aliquoting heuristic is construct a natural graph according to the definition below.

**Definition 4 ***Let A be a duplicated genome. A *natural graph *NG*(*V, E*) *is a graph whose vertices V are the adjacencies and telomeres of A and each extremity is connected to all of its paralogous extremties by an edge in the set E*.

**Figure 3:**
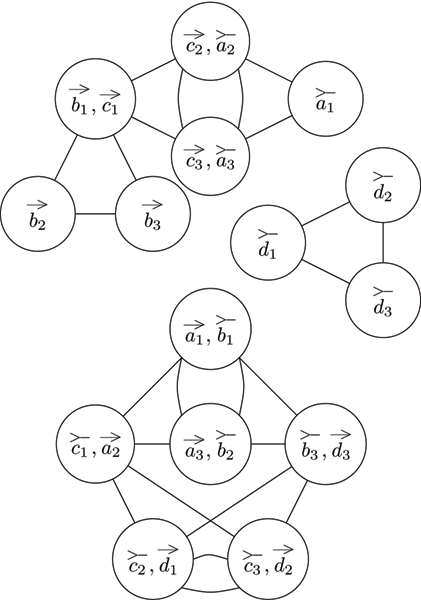
**Natural graph**. A natural graph describing the genome depicted in Figure 1a. Observe that the genome has a ploidy of 3 and every vertex in the natural graph has a degree of either 2 or 4.

Observe that, with this definition, the vertices have a degree of either *p *- 1 or 2 (*p *- 1) where *p *is the ploidy of the genome. Thus, for the interesting case of *p *≥ 3, every vertex has a degree of at least two (see Figure 3 for an example). However, essential to genome halving is that every vertex in the natural graph have a degree of at most two.

A graph with a degree of at most two is important for two reasons. Firstly, it allows for the maximum cardinality set of compatible adjacencies and telomeres of the natural graph to be easily computed, as in [2], or for the double cut and join operation to be applied directly on the graph, as in [4]. Secondly, it allows for the cycles and paths of the natural graph to be trivially computed.

Since the heuristic must handle graphs with vertices of degree three or more, it must find this information through some other means. Hence, while the natural graph is still the base of the solution, a great deal of additional work is needed to extract the relevant information from it.

### Generate modified clique graphs

While many useful properties of the natural graph are obfuscated when the ploidy of the graph is increased to three or more, one property is still very clear: the graph's cliques. Every extremity of the genome corresponds to one clique in the natural graph. Thus, to capitalize on this, we create a clique graph from the natural graph according to the following definition.

**Definition 5 ***Let NG*(*V, E*) *be a natural graph constructed from a genome A. A *clique graph *CG*(*V', E'*) *is a graph whose vertices V' are the extremities of A and there exists an edge *{*u, v*} *in the set E' if there exists an adjacency *{*u*_*x*_, *v*_*y*_} *in A where *1 ≤ *x, y *≤ *p and p is the ploidy of A*.

Observant readers may notice that, in order to compute the clique graph, the natural graph is not needed. It is included in the pseudocode and the above section to help stress the link between the genome aliquoting heuristic and genome halving algorithm and to better explain the origin of the clique graphs, but it is not necessary in an actual implementation.

**Figure 4:**
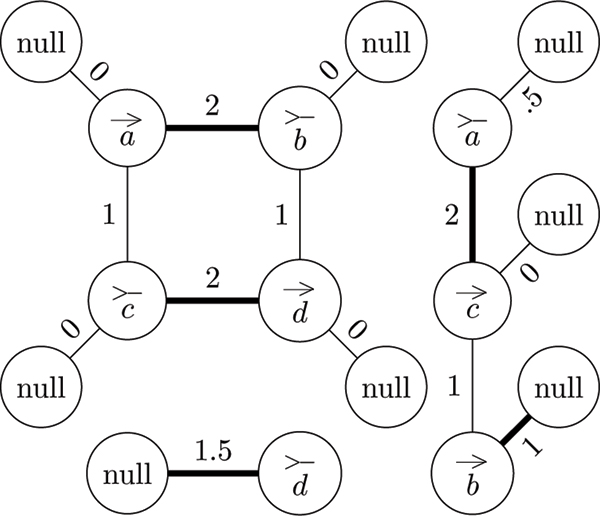
**Modified clique graph**. A modified clique graph describing the natural graph depicted in Figure 3. The five bold edges represent all the edges that belong to the maximum weight matching of this graph.

The clique graph as defined above is not sufficient for our purposes. It is missing the data concerning the telomeres contained in the natural graph. To solve this problem, the algorithm must modify the clique graph by creating null vertices, one for each vertex of the clique graph, and connecting each null vertex to its corresponding non-null vertex by an edge (see Figure 4).

Now, after modifying the clique graph, each edge corresponds to either an adjacency or telomere in the genome. However, it would be useful to know how many adjacencies and telomeres correspond to each edge. The solution is to weight the edges. If the edge corresponds to an adjacency, assign it a weight equal to the number of adjacencies to which it corresponds. If the edge corresponds to a telomere, assign it a weight equal to *half *the number of telomeres to which it corresponds (see Figure 4). The reason why telomeres are weighted half as much as adjacencies is because telomeres are weighted half as much as adjacencies when computing the double cut and join distance. Since the objective is compute double cut and join distance directly from the clique graph, it is important to capture this detail.

Even with all the additional information added to the clique graph some information is still missing. In particular, adjacencies of the form {*u*, *u*} are not considered in this graph. This is intentional. Such information doesn't help in the aliquoting of the graph so the algorithm simply ignores it.

### Compute maximum weight matching

The process of aliquoting a genome is in theory quite simple. For each extremity simply select one adjacency or telomere from the rearranged duplicated genome and add it to the perfectly duplicated genome, copying it as many times as needed to get the necessary ploidy. The difficulty of finding a parsimonious perfectly duplicated genome lies in the copies. If the copies are not compatible with the selected adjacency or telomere then the distance between the original genome and the constructed genome increases. Thus, any algorithm that endeavors to construct the most parsimonious perfectly duplicated genome must maximize the number of compatible adjacencies and telomeres. Thus, as in the genome halving problem, one important objective of the genome aliquoting problem is to find the maximum cardinality set of compatible adjacencies and telomeres. Using the modified clique graph, it is now possible to do just that.

Observe that a pair of adjacencies and/or telomeres is compatible if their corresponding edges in the modified clique graph do not share a vertex in common (we say the edges are *independent*) or if they are represented by the same edge. Thus, to find the maximum cardinality set of compatible adjacencies and telomeres the algorithm must find a set of independent edges with maximum weight. Consider the following well-known graph problem:

**Definition 6 ***The *maximum weighted matching problem *is defined as follows: find a set of independent edges such that the sum of the weights of the edges is maximum*.

The maximum weighted matching problems was famously solved by Edmonds in polynomial time [7]. Thus, it is possible to compute the maximum set of compatible adjacencies and telomeres in polynomial time. Hence, the algorithm has recovered the first piece of data that was obfuscated when the transition to genome with ploidy of three or greater was made (see Figure 4 for an example).

### Find cycle and paths

Typically, a single double cut and join operation reduces the distance by one. However, occasionally a double cut and join operation reduces the distance by two instead. In the genome halving problem, it was possible to detect the double cut and join operations that would reduce the distance by two by detecting the *even *cycles (even in terms of number of edges) and *odd *paths in the natural graph. Since the vertices of the clique graph are the edges of the natural graph and the edges of the clique graph are the vertices of the natural graph, an even length cycle in the clique graph corresponds to an even length cycle in the natural graph but an *even *length path in the clique graph corresponds to an *odd *length path in the natural graph. Thus, the genome aliquoting heuristic must find even length cycles and even length paths in the clique graph in order to detect the double cut and join operations that reduce the distance by two.

**Figure 5:**
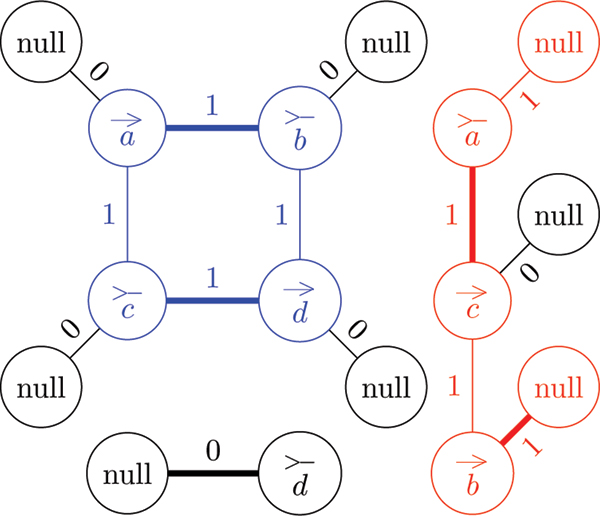
**Re-weighted modified clique graph**. A modified clique graph depicted in Figure 4, after it has been re-weighted for cycle and path detection. Red edges and vertices represent the edges and vertices along an even length alternating path. Blue edges and vertices represent the edges and vertices along an even length alternating cycle. As can be seen from the figure, this graph has exactly one even alternating cycle and exactly one even alternating path each with a flow of 1.

When detecting cycles and paths for the genome aliquoting problem there is are two additional details that must be considered over the detecting cycles and path for the genome halving problem. First, unlike the genome halving problem, not every cycle and path is a component of the natural graph. For cycles, this doesn't change anything. But for paths, this means that there is an additional detail that must be considered. For the purposes of the genome aliquoting problem, a path is any path between two telomeres in the natural graph meaning that it is between two null vertices in the modified clique graph (see Figure 5).

Secondly, an edge in the clique graph may correspond to several edges in the natural graph. In the clique graph this is represented by the weights of the edges. Thus, it is important to account for the weights when detecting cycles and paths.

Because the task has changed from finding the matching to finding the cycles and paths the weights of the clique graph need to be modified slightly as well. In order to understand why and how to modify the edges, it is important to understand what both the matching and the cycles and paths represent.

In the genome aliquoting problem, the goal is to *construct *another genome that minimizes the distance. Thus, the algorithm must build a genome in such a way that the fewest number of double cut and join operations need to be performed. Another way of looking at it is that we are "pre-performing" double cut and join operations; any double cut and join operation that the algorithm can legally perform at this stage is one fewer double cut and join operation that it must perform later. The edges of the matching, and their corresponding edge weights, indicate the double cut and join operations that can be legally performed during the creation of the genome, *i.e. *they indicate the double cut and join operations that need *not *be performed when computing the distance.

When computing the cycles and paths, the algorithm is no longer "pre-performing" double cut and join operations. Rather, the algorithm is attempting to predict the double cut and join operations that will be performed when computing the distance between the original genome and the constructed genome. Thus, while the weights of unmatched edges are still correct as the extremities represented by those edge will need to be moved when the distance is computed, the weights of the matched edges are not correct as the double cut and join operation indicated by those weights will have already have been performed.

Therefore, the algorithm must change the weights of the matched edges to reflect the situation during the calculation of the distance.

So what, if anything, do the matched edges represent while the distance is being computed? The matched edges represent the final state of two extremities; the extremities that correspond to the vertices that the edge connects. The current weight of the matched edge indicates how many of the pairs of extremities have reached that final state. Therefore, the complement of that weight indicates how many pairs of extremities have not yet reached that final state. Hence, if *p *is the ploidy of the graph and *w*(*e*) is the weight of a matched edge *e*, then *w*(*e*) = *p *- *w*(*e*).

Unfortunately, there is a complication when it comes to re-weighting the graph. In the original weighting scheme, the weights of edges connected to a null vertex where weighted half as much as those connect to two non-null vertices. The algorithm needs to re-weight the edges to be on par with the others. Hence, if *e *is a matched edge connected to a null vertex then its weight should be *w*(*e*) = *p *- 2·*w*(*e*) and if *e *is an *unmatched *edge connected to a null vertex then its weight should be *w*(*e*) = 2·*w*(*e*). See Figure 5 for an example on how to re-weight the graph.

Once the algorithm has updated the weights to reflect the change in objective how do the weights factor into the detection of cycles and paths? It is easy to see that each weight represents an upper bound on some kind of resource. The matched edges represent an upper bound on the double cut and join operations used in computing the distance and the unmatched edges represent an upper bound on the resources available to the double cut and join operations, *i.e. *the extremities. Thus, matched and unmatched edges, each in a different way, represent an upper bound on the number of double cut and join operations.

In the genome halving problem, any sequence of double cut and join operations that form an even cycle or an even path produces an extra double cut and join operation. It is the same for the genome aliquoting problem except that a cycle or path may represent multiple sequence of double cut and join operations and, hence, multiple extra double cut and join operations. As each edge represents an upper bound on the number of double cut and join operations that can act on that edge, the number of sequences of double cut and join operations is the smallest edge weight on the even cycle or even path. Therefore, it is the *flow *of the even cycle or even path that the algorithm must compute.

There is another factor that the algorithm must account for when choosing cycles and paths: they must *alternate *between matched and unmatched edges. As mentioned before, matched edges represent the double cut and join operations and unmatched edges represent the extremities used in those double cut and join operations, thus, both are needed. By alternating between matched and unmatched edges, we learn which extremities must be matched to which double cut and join operations in order to produce an extra double cut and join operation.

The authors of this paper make no assertion about the best method to determine the cycles and paths other than that any implementation of this algorithm should make an effort to maximize the number of cycles, and their flow, and the number of paths and their flow. Ideally this should be done in one step, but, as this is a heuristic, doing it in two steps (finding cycles followed by path or *visa versa*) is possible but with a potential lose in accuracy. Unfortunately, because of the simplicity of the later method, it is the method that we use in our implementation (see section Implementation below).

Create genome

**Figure 6:**
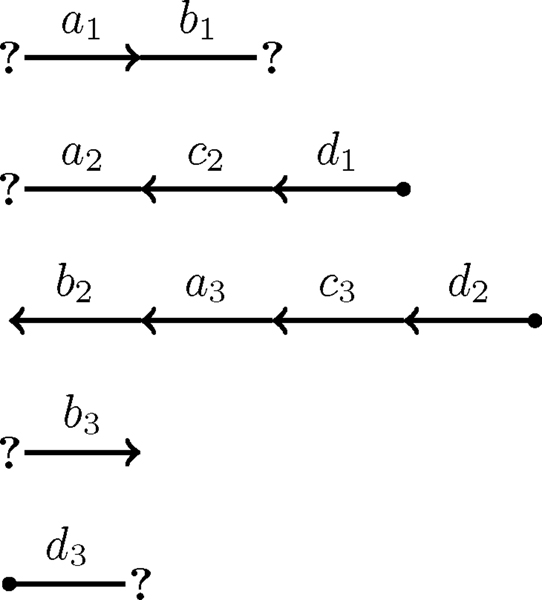
**Compatible set**. A partially aliquoted genome derive from the maximum cardinality set of compatible adjacencies and telomeres that was derived from the matching depicted in Figure 4.

At this stage it is now possible to construct a perfectly duplicated genome. As both the genome and the maximum cardinality set of compatible adjacencies and telomeres are sets of adjacencies and telomeres, the maximum cardinality set of compatible adjacencies and telomeres can be used to immediately create part of the perfectly duplicated genome as depicted in Figure 6.

For the remaining adjacencies and telomeres of the perfectly duplicated genome, the matched edges that are connected to two non-null vertices indicate the adjacencies and those that are connected to only one non-null vertex indicate the telomeres. However, pairing these extremities alone does not guarantee a good aliquoting. In some cases it is important to know the subscripts of the extremities to be paired.

**Figure 7:**
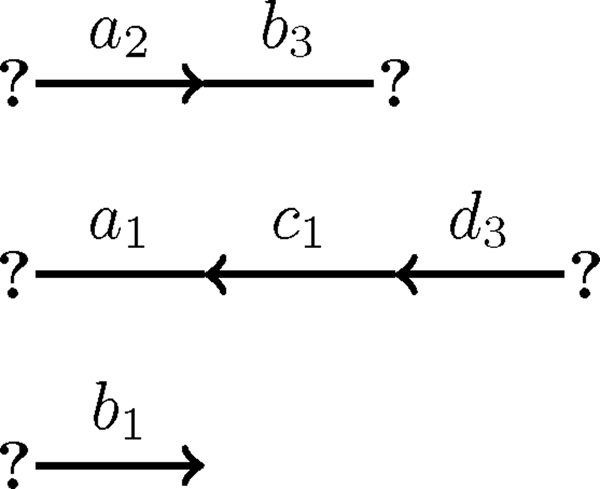
**Joining along the paths and cycles**. A partially aliquoted genome derived from the path and cycle depicted in Figure 5.

To get this additional information, the algorithm must *join along *the cycles and paths that it detected (see Figure 7). To do this, take the two adjacencies represented by two unmatched edges along a cycle or path (one adjacency from each unmatched edge) and use their adjacent matched edge as a template to join them. For example, assume without a loss of generality let {*a*, *b*} be a matched edge along one of the detected cycles. This edge will be adjacent to two unmatched edges. Each unmatched edge will represent some adjacencies in the original genome, say {*c*_*x*_, *a*_*y*_} and {*b*_*z*_, *d*_*w*_}. To join along the cycle the algorithm adds the overlap between the matched edge and its adjacent unmatched edges to the constructed genome, hence it adds {*a*_*y*_, *b*_*z*_}.

When it comes to paths there are two exceptions to the above rule as there will be two edges in the path that are connected to a null vertex, one matched edge and one unmatched edge. Recall that null vertices correspond to telomeres. Consider the unmatched edge a telomere for the purpose of joining along the path. Since it will overlap with its neighboring matched genome, there is no other difference. As for the matched edge, the algorithm should simply ignore it.

**Figure 8:**
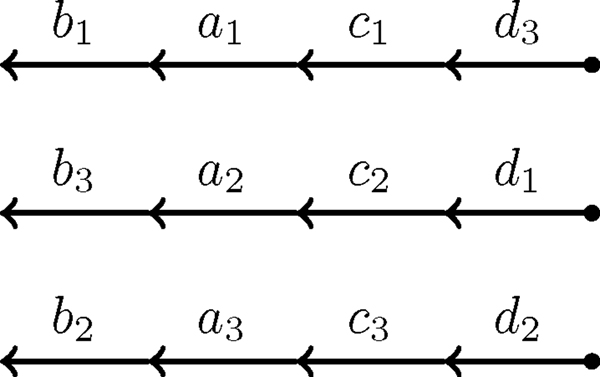
**A perfectly duplicated genome**. A perfectly duplicated genome created by combining the partially aliquoted genomes in Figures 6 and 7. In this particular case, no random joinings were needed to complete the genome.

After joining along the cycles and paths, it is possible to combine that result with the maximum cardinality set of adjacencies and telomeres to create the genome. Figure 8 is an example of this. However, while creating the genome there may be some ambiguity. For example, if a cycle or path has a flow of two then there will be two possible extremities that could be joined at each edge. Another common occurrence of ambiguity is the case where some extremities are neither part of the maximum cardinality set of adjacencies and telomeres nor are they identified while joining along the cycles and paths.

All cases of ambiguity are resolved in the same manner: arbitrarily combining the extremities. In experiments arbitrarily combining the ambiguous extremities never increased or decreased the distance. In fact, we conjecture that it can be proven that it never will.

### Implementation

Our implementation of the heuristic follows the pseudo-code above fairly closely. Detecting the cycles and paths are performed independently, with the cycles being detected first since they are usually a greater contributer to reducing the distance.

We use an implementation of the Edmonds algorithm to exactly compute the maximum weight matching problem [7] as well as a slightly modified implementation of the Edmonds-Karp maximum flow algorithm [8] to find the flow of the alternating even paths.

For the alternating even cycles, we use a greedy heuristic that simply tries to find the smallest cycles (in terms of number of edges) in hopes that this will produce the least conflict and therefore the maximum flow. Detecting the cycles is the only heuristic used in our heuristic but even if we had used an exact algorithm for this step our algorithm would still only be a heuristic.

Our implementation of the heuristic runs in time polynomial to the number of genes in the genome. It performs extremely quickly requiring very large genomes to produce any kind of noticeable slowdown.

## Results and Discussion

To test the heuristic, we ran it on some simulated data. Unfortunately, it is impossible to generate a genome with a known rearrangement distance. Thus, we generated genomes that were small enough that we could retrieve the exact result using a brute force algorithm. We performed tests on seven types of genomes: hexaploids (three copies of each gene) with two, three and four genes and octoploids (four copies of each gene) with two and three genes. We randomly generated 25 examples of each of the seven types of genomes and ran both our heuristic and a brute force algorithm and compared the solutions.

We divide the results from our experiment into four categories: exact, minor inexact, major inexact and error. The category exact means that our algorithm got the same result (in terms of distance) as the brute force algorithm.

We can trivially assume that the distance must be less than or equal to *n*·(1 - *p*) where *p *is the ploidy of the genome and *n *is the number of genes. Thus, a minor inexact result means that our algorithm did better than the trivial case but worse than the brute force algorithm.

There are two types of major inexact results. The first is the case where the heuristic produced a distance that was *lower *than the lowest possible result (the result of the brute force algorithm). In Table 1, this type of major inexact result is indicated by the number before the slash. The second is the case where the heuristic produced a distance that was equal to the trivial case and this was not the best result (*i.e. *the brute force algorithm returned a lower result). In Table 1, this is indicated by the number after the slash. The heuristic never returned a result that was worse than the trivial result.

**Table 1:** Heuristic/brute force comparison. The results of our experiment comparing our heuristic to a brute force algorithm.

Test Case	Exact	Minor Inexact	Major Inexact	Error
**Hexaploid**				
2 genes	19(16)	2	3(0)/1	6
3 genes	9(6)	11(9)	1(0)/4	6
4 genes	5(3)	17(14)	2(0)/1	7

**Octaploid**				
2 genes	14(12)	9(7)	1(0)/1	5
3 genes	6(4)	16(12)	2(0)/1(0)	9
TOTAL	53(41)	55(44)	9(0)/8(7)	33

The final category, error, indicates the number of times that the algorithm returned a genome that was not properly aliquoted. Any result in this category always occurs in conjunction with a result from another category. Thus, if we consider all the error results to be "contaminated" and unusable, we indicate what the actual result for each of the other categories would be in parenthesizes.

Since the first test of the heuristic could only be conducted on smaller genomes, we conducted a second test of the heuristic on larger genomes but without the brute force algorithm. We compared the results of this second test with the trivial case to indicate that our heuristic returns a non-trivial result. This time, not only did we aliquote hexaploids and octaploids, but also larger polyploids, specifically decaploids (five copies of every gene) and icosaploids (10 copies of every gene). We also studied our algorithm on genomes with more genes, starting at 40 genes in the smallest case and 100 genes in the largest. Again we attempted our algorithm on 25 randomly generated examples of each genome.

For this set of experiments we simple checked to see if the heuristic returned a distance less than, equal to, or greater than the trivial case. Additionally, we checked to make sure that the genome was properly aliquoted. The results of this experiment are listed in Table 2.

**Table 2:** Heuristic on large genomes. The results of our experiment testing the performance of the heuristic on large genomes.

Test Case	Less Than	Equal To	Greater Than	Error
**Hexaploid**				
40 genes	25(19)	0	0	6
60 genes	25(18)	0	0	7
80 genes	25(19)	0	0	6
100 genes	25(19)	0	0	6

**Octaploid**				
40 genes	25(19)	0	0	6
60 genes	25(19)	0	0	6
80 genes	25(20)	0	0	5
100 genes	25(22)	0	0	3

**Decaploid**				
40 genes	25(17)	0	0	8
60 genes	25(22)	0	0	3
80 genes	25(20)	0	0	5
100 genes	25(19)	0	0	6

**Icosaploid**				
40 genes	25(22)	0	0	3
60 genes	25(23)	0	0	2
80 genes	25(24)	0	0	1
100 genes	25(23)	0	0	2

TOTAL	400(325)	0	0	75

Finally, we decided to test our algorithm on real, rather than simulated, data. We applied our heuristic to the hexaploid wheat that we retrieved from the Gramene database [9]. Note that after *p*-ploidization, many of the gene families thus created are reduced by the various processes of gene loss. Eventually, after a long time has elapsed, very few of the genes will have retained *p *copies. Thus, in the wheat data we could only identify 92 gene families with three members. On this input, our heuristic returned an aliquoted genome with a distance of 138 but with one incorrectly aliquoted chromosome. Correcting that chromosome would produce an aliquoted genome with a distance of 139, well below the trivial distance of 184. The corrected result had a total of 45 chromosomes of which 3 were circular.

There are several interesting results from the experiments. Let's begin by examine the negatives.

21% of the time, the heuristic returned an improperly aliquoted genome. On the smaller genomes this was higher (26%) whereas on the larger genomes this was lower (19%). The reason why the algorithm gives improperly aliquoted genomes goes back the genome halving papers.

In [1], it was stated that, if *C*_1 _and *C*_2 _were two identical chromosomes, then a circular unichromosomal genome where *C*_1 _and *C*_2 _are concatenated together at both end points can be considered an acceptable halving. When double cut and join was introduced, it was considered desirable to allow circular chromosomes so an effort was made to generalize this statement. In the first attempt, in [2], any concatenation of two halved chromosomes, with one or both endpoints, in either a unichromosomal or multi-chromosomal setting was considered acceptable. [4] modified this definition by adding the restriction that both endpoints needed to be joined.

In our genome aliquoting heuristic, we used the definition from [4]. However, even though this definition is more restrictive than [2], it creates a problem in the generalized aliquoting case (not in the halving case). To see the problem consider a hexaploid with three aliquoted chromosomes *C*_1_, *C*_2 _and *C*_3_. The problem is that, because we have more than two parts, it might occur that *C*_1 _and *C*_2 _concatenate to form *C*_1_*C*_2 _but *C*_3 _does not join that concatenation. This is an improperly aliquoted genome, but, so long as all the chromosomes are circular, it is a proper aliquoting according the definition of valid. Hence, the heurisitic accepts it. All the errors listed above are genomes of this form.

However, this error is not particularly severe as it is easy to detect and it is easy to correct. For each problem case that occurs it will take at most two double cut and join operations to correct so we can simply increase the distance by that amount. This becomes particularly important when we consider the second major problem with our algorithm, the "better than optimal" results that where produced in some cases.

As we can see from Table 1, the "better than optimal" results only occur in conjunction with an invalid aliquoting. The increase in distance that comes from correcting such an aliquoting will invariably also correct the distance, although it may not yield the exactly optimal distance (it might be slightly worse).

However, perhaps the most serious problem is that indicated on Table 1, in the row "Octoploid with 3 genes" and the column "Major Inexact". The result after the slash indicates a case where the heuristic produces a trivial result and an incorrectly aliquoted genome at the same time. This means that it is possible for the heuristic to produce an incorrectly aliquoted genome that cannot be fixed. Fortunately, this seems to be an unlikely occurrence, especially on larger genomes.

There are, however, many positive attributes of the heuristics as well. As seems to be indicated by the case of icosaploids, the number of incorrect aliquotings seem to go down on very large genes. This is likely because the heuristic has a bias towards selecting adjacencies in the original genome that tend to produce circular chromosomes. But, with a larger genome the heuristic will select more telomeres and, thus, produce more linear chromosomes. Even selecting few telomeres in proportion to the number adjacencies seems to dramatically reduce the number of circular chromosomes. And, since the improper aliquoting only occurs with circular chromosomes, the chance of an improper aliquoting occuring is reduced.

Additionally, we can see that, on larger genomes, the heuristic performs better than the trivial case. In these cases the heuristic tended to give a distance that was about 15% lower than the trivial case. Finally, at least on the smaller genomes, we can clearly see that most of the time the heuristic gave a good result. 33% of the results are the correct answer without any error and another 35% of the results have a better than trivial answer without any error. We can also see that in most of the cases where there was an error it could be corrected for and the result would still be better than trivial.

## Conclusion

From the results we can conclude that the algorithm seems to perform very well as a heuristic for the genome aliquoting problem. We have shown that in small cases, the algorithm performs very close to optimal and, while it is easy to imagine that the error ratio increases as the genome gets larger the algorithm never-the-less continues to perform significantly better than any trivial case.

While the heuristic has been known to occasionally produce a genome with a "better than optimal" distance, this is always the product of an improperly aliquoted genome, and, thus, it is easy to detect and fix. Furthermore, fixing such a genome will also correct the distance. Thus, users concerned about such a result can easily implement a post-processing step that tests for this problem and corrects it when found.

Thus, we conclude that the algorithm produces a reasonably parsimonious solution for any instance of the genome aliquoting problem.

There are a number of improvements that can be done on this heuristic. A better method for detecting cycles and paths and maximizing their flow is needed. Ideally, such a method should be able to detect both cycles and paths simultaneously and it should produce an exact result.

Additionally, a better definition of validity is needed. Because we wanted to include circular chromosomes, the definiton from [4] was used. However, by allowing certain types of circular chromosomes the problem of "better than optimal" solutions was introduced. The El-Mabrouk and Sankoff definition [1] would eliminate circular chromosomes and, therefore, this problem.

Finally, perhaps the most important objective for future work is to examine the possible existence of an exact algorithm. Given how close this problem is to the median problem [10], it may be that his problem is NP-complete [11]. On the other hand, given how well this heuristic performs it may be that a polynomial time algorithm exists. We conjecture that if the above improvements to the heuristic can be made and that an algorithm can be found that finds the maximum weight matching and the cycle flow and path flow all at the same time then the algorithm will return an exact solution.

## Competing interests

The authors declare that they have no competing interests.

## Authors' contributions

RW devised the algorithms and did the mathematical analysis. He also drafted the paper. DS contributed to the research strategy, participated in discussions throughout the research and revised the manuscript.
